# Dr. Hamilton D. Wey

**Published:** 1909-04

**Authors:** 


### OBITUARY

DR. HAMILTON D. WEY, of Elmira, N. Y., died March 16, 1909, at Callao, Peru, whither he went sometime ago, for the benefit of his health, aged 54 years. He was the only son of the late Dr. William C. Wey, himself a leading member of the medical profession in this state. The son, the subject of this memoir, received his medical degree from the New York University Medical College in 1878, and soon afterward became associated with his father in the practice of medicine, and so continued until the death of the latter in 1897. Since that time Dr. Hamilton D. Wey has been recognised as one of the foremost practitioners in the Southern Tier of counties.

Dr. Wey was elected president of the Medical Society of the State of New York in January, 1904, and presided at the annual meeting held in January, 1905. During this year,—namely, in May, 1904, he lost his only son, a bright little fellow of about four years, a sad blow to both parents. He was a member of the American Medical Association of the Elmira Academy of Medicine of which he had been president: consulting physician to the Arnot-Ogden Memorial Hospital, and a member of several other organisations including the Elmira Club.

Dr. Wey, in addition to his superb professional equipment, was, like his father, a man of thorough training, a citizen of worth, and a most companionable man in every way. His loss will be deeply felt, not only in Elmira, but throughout the State.

